# Efficacy and safety of sintilimab plus doxorubicin in advanced soft tissue sarcoma: A single-arm, phase II trial

**DOI:** 10.3389/fphar.2022.987569

**Published:** 2022-10-06

**Authors:** Zhichao Tian, Shuping Dong, Wenli Zuo, Po Li, Fan Zhang, Shilei Gao, Yonghao Yang, Chao Li, Peng Zhang, Xin Wang, Jiaqiang Wang, Weitao Yao

**Affiliations:** ^1^ Department of Bone and Soft Tissue, The Affiliated Cancer Hospital of Zhengzhou University, Zhengzhou, Henan, China; ^2^ Department of Hematology, The Affiliated Cancer Hospital of Zhengzhou University, Zhengzhou, Henan, China; ^3^ Department of Immunotherapy, The Affiliated Cancer Hospital of Zhengzhou University, Henan, Zhengzhou, China

**Keywords:** PD-1 inhibitor, chemotherapy, chemoimmunotherapy, undifferentiated pleomorphic sarcoma, dedifferentiated liposarcoma

## Abstract

**Background:** Chemoimmunotherapy is safe and efficacious in treating many types of malignant tumors. However, clinical data demonstrating the effect of this combination treatment in patients with metastatic soft tissue sarcoma (STS) are currently limited. This study evaluated the safety and efficacy of a programmed cell death protein 1 (PD-1) inhibitor plus doxorubicin in patients with advanced STS who failed previous systemic therapy.

**Methods:** This was a single-center, single-arm, open-label phase II trial. Patients with unresectable or metastatic STS who had previously failed systemic therapy were enrolled. Patients received up to six cycles of doxorubicin and sintilimab (a PD-1 inhibitor), while sintilimab treatment continued for up to 2 years. Primary outcomes were objective response rate (ORR) and safety. Univariate Cox proportional hazards model was used to analyze the relationship between clinicopathological parameters and progression-free survival (PFS).

**Results:** A total of 38 patients (20 men and 18 women) were enrolled in this study. The overall ORR was 39.5%, disease control rate was 71.1%, and the median PFS was 4.5 months [95% confidence interval (CI), 3.0–8.5 months]. The adverse events (AEs) associated with the combined treatment were mild, manageable, and well-tolerated. The most common grade 3 or higher AEs were hematologic, including leukopenia (21.1%), anemia (18.4%), and thrombocytopenia (18.4%). Patients with undifferentiated pleomorphic sarcoma (UPS) or dedifferentiated liposarcoma had a significantly longer PFS than those with other pathological subtypes [hazard ratio (HR) = 0.42, 95% CI 0.21–0.83; *p* = 0.013]. There was no significant difference in the median PFS between patients who had previously received anthracycline-based chemotherapy and those who had not (HR = 0.74, 95% CI 0.34–1.58, *p* = 0.43).

**Conclusion:** Sintilimab plus doxorubicin is a safe and promising treatment for patients with advanced STS who have failed previous systemic therapy (including anthracycline-based chemotherapy). The efficacy of this combination therapy in UPS and dedifferentiated liposarcoma is superior to that in other sarcomas.

**Clinical Trial Registration:** https://www.chictr.org.cn, registration number: ChiCTR1900027009.

## Introduction

Although considered a rare malignant disease, more than 200,000 new cases of soft tissue sarcoma (STS) are diagnosed worldwide each year (Yang et al., 2019). More than half of the patients with STS eventually progress to an advanced stage (Gamboa et al., 2020). The conventional first-line treatment for advanced STS is anthracycline-based chemotherapy, which has a response rate of less than 20% and a median progression-free survival (PFS) of less than 6 months (Seddon et al., 2017; Tap et al., 2017; Gamboa et al., 2020). Gemcitabine-based regimen is often considered as second-line treatment after anthracycline, with an expected median PFS similar to that of anthracyclines (von Mehren et al., 2022). Multi-target tyrosine kinase inhibitors (TKIs) have also been shown to be effective against selective STS although they have a lower median PFS than anthracycline-based and gemcitabine-based regimens (Kyriazoglou et al., 2022). The low response rates of these treatments result in a median overall survival of less than 2 years for advanced STSs (Seddon et al., 2017; Tap et al., 2017). Therefore, there is a need for new and effective treatment of advanced STS.

The clinical application of programmed cell death protein 1 (PD-1) inhibitors has brought remarkable progress in treating various malignant diseases (Khan et al., 2021). However, the response rate of PD-1 inhibitor monotherapy in STS is approximately 8% (Roulleaux Dugage et al., 2021; Meyer, 2022). To improve the efficacy of immunotherapy, PD-1 inhibitors have been used in combination with chemotherapy and other therapies (Dajsakdipon et al., 2022; Kerrison et al., 2022). Several studies have demonstrated the potential synergistic effect of the PD-1 inhibitor and doxorubicin combination in the treatment of advanced STS (Pollack et al., 2020; Livingston et al., 2021).

Sintilimab is a PD-1 inhibitor marketed in China (Hoy, 2019). In our previous study, its safety and promising antitumor activity in STS were confirmed (Tian et al., 2022). Recent clinical trials have shown that sintilimab combined with chemotherapy is more effective than chemotherapy alone in advanced esophageal, gastric, and lung cancers (Yang et al., 2021; Zhou et al., 2021; Jiang et al., 2022; Lu et al., 2022). However, no clinical trials of sintilimab in combination with chemotherapy in STS have been reported. In this study, we report the results of a single-center phase II clinical trial of sintilimab plus doxorubicin as the second- or later-line treatment of advanced STS.

## Methods

### Patients and eligibility criteria

This was an open-label, single-center, single-arm phase II trial that assessed the effects of sintilimab combined with doxorubicin in two or more lines of therapy for advanced STS. The main inclusion criteria were: 1) patients aged ≥18 years; 2) histologically confirmed STS; 3) locally advanced or metastatic disease; 4) previously received at least one line of systemic therapy; 5) measurable and progressive disease at recruitment according to the response evaluation criteria in solid tumors (RECIST; version 1.1); 6) Eastern Cooperative Oncology Group performance status of 0–2; and 7) adequate hematological, hepatic, renal, and metabolic functions. The complete inclusion criteria are provided in the online protocol (https://www.chictr.org.cn).

This study was approved by the Ethics Committee of Henan Cancer Hospital and registered in the Chinese Clinical Trial Registry (https://www.chictr.org.cn, registration number: ChiCTR1900027009). The trial was performed in accordance with Good Clinical Practice and the Declaration of Helsinki. Written informed consent was obtained from all the patients.

### Treatment protocol

Patients were treated with doxorubicin (35 mg/m^2^ per day via intravenous bolus) on days 1 and 2 and sintilimab (200 mg *via* a 30-min intravenous infusion) on day 4 of a 21-days cycle for up to six cycles unless progressive disease (PD) or unacceptable adverse events (AEs) occurred. After cycle 6, sintilimab treatment was continued for up to 2 years unless there was PD or unacceptable AEs. The study permitted two dose-reduction levels of doxorubicin, from 35 mg/m^2^ to 30 mg/m^2^ to 25 mg/m^2^. Sintilimab dose was not reduced but was delayed when AEs occurred. When the patients developed unacceptable AEs, the treatment was delayed until recovery. PEGylated recombinant human granulocyte-colony stimulating factor was routinely administered after each combination treatment. Dexrazoxane was routinely used to prevent cardiotoxicity in patients administered a cumulative doxorubicin dose higher than 300 mg/m^2^.

### Evaluation and outcomes

Efficacy was assessed according to RECIST (version 1.1). Safety was evaluated according to the National Cancer Institute’s Common Terminology Criteria for Adverse Events (version 4.0). Target lesion was assessed using a computed tomography scan performed within 28 days prior to the initiation of baseline treatment. Scans were performed every 6 weeks (or immediately until there was a clear sign of PD) during the study period until the first documented PD or start of new anticancer therapy. In our study, a 9-weeks response evaluation was performed.

The primary outcomes were safety and objective response rate (ORR). Secondary outcomes included disease control rate (DCR) and progression-free survival (PFS). ORR was defined as the sum of the complete response (CR) and partial response (PR) rates. DCR was defined as the sum of ORR and stable disease rate. PFS was defined as the time from the start of the treatment protocol to the first occurrence of PD or death.

### Statistical analyses

Statistical software SAS (version 9.4) was used for data analysis. Descriptive statistics were used to summarize the subjects’ characteristics, treatment status, and drug safety characteristics. The measurement data are described in terms of the number of cases, mean, percentage, standard deviation, maximum, minimum, and median. PFS was estimated using the Kaplan-Meier method. A univariate Cox proportional hazards model was used to analyze the relationship between the clinicopathological parameters and PFS. The corresponding figures were generated using GraphPad Prism (version 5.0). All statistical analyses were two-sided, and a *p* value of <0.05 was considered statistically significant. The data analyzed were collected between December 2019 and March 2022.

## Results

### Patient characteristics

In this phase II trial, 38 patients (20 men and 18 women) with advanced STS were enrolled, and two patients (5.3%) remained on sintilimab therapy as of the data cutoff date. The average age was 47.61 ± 12.06 years. The most common histological subtypes were undifferentiated pleomorphic sarcoma (UPS) (n = 16, 42.1%), synovial sarcoma (n = 4, 10.5%), dedifferentiated liposarcoma (n = 3, 7.9%), and leiomyosarcoma (n = 3, 7.9%). All patients had previously received 1–3 lines of systemic therapy, and 28 (73.7%) had previously received anthracycline-based chemotherapy. In addition, gemcitabine-based chemotherapy, TKIs, and albumin-bound paclitaxel had also been used in front-line treatment. The demographic and clinical data, including disease-related factors, are presented in Table 1.

**TABLE 1:** Clinical characteristics of enrolled patients.

Characteristics	No. (%) (n = 38)
Gender	
Male	20 (52.6%)
Female	18 (47.4%)
Age	47.61 ± 12.06
ECOG PS	
0	17 (44.7%)
1	18 (47.4%)
2	3 (7.9%)
Histology	
Undifferentiated pleomorphic sarcoma	16 (42.1%)
Synovial sarcoma	4 (10.5%)
Dedifferentiated liposarcoma	3 (7.9%)
Leiomyosarcoma	3 (7.9%)
Myxofibrosarcoma	2 (5.3%)
Myxoliposarcoma	2 (5.3%)
Angiosarcoma	2 (5.3%)
Epithelioid sarcoma	2 (5.3%)
Rhabdomyosarcoma	1 (2.6%)
MPNST	1 (2.6%)
Spindle cell sarcoma	1 (2.6%)
Clear cell sarcoma	1 (2.6%)
Stage	
IV	38 (100.0%)
Primary site	
Extremities	21 (55.3%)
Trunk	9 (23.7%)
Head	3 (7.9%)
Retroperitoneal	3 (7.9%)
heart	2 (5.3%)
Metastatic site	
lungs	31 (81.6%)
other	7 (18.4%)
Lines of previous systemic therapy	
1	18 (47.4%)
2	17 (44.7%)
3	3 (7.9%)
Regimens used in prior lines	
Anthracycline-based regimens	28 (73.7%)
Gemcitabine-based regimens	14 (36.8%)
TKIs	10 (26.3%)
Albumin-bound paclitaxel	8 (21.1%)

Data are presented as numbers (percentages) or means ± standard deviations. Abbreviations: ECOG PS, Eastern Cooperative Oncology Group performance status; MPNST, malignant peripheral nerve sheath tumor. TKIs, tyrosine kinase inhibitors, including anlotinib, apatinib, and pazopanib.

### Efficacy

**FIGURE 1:**
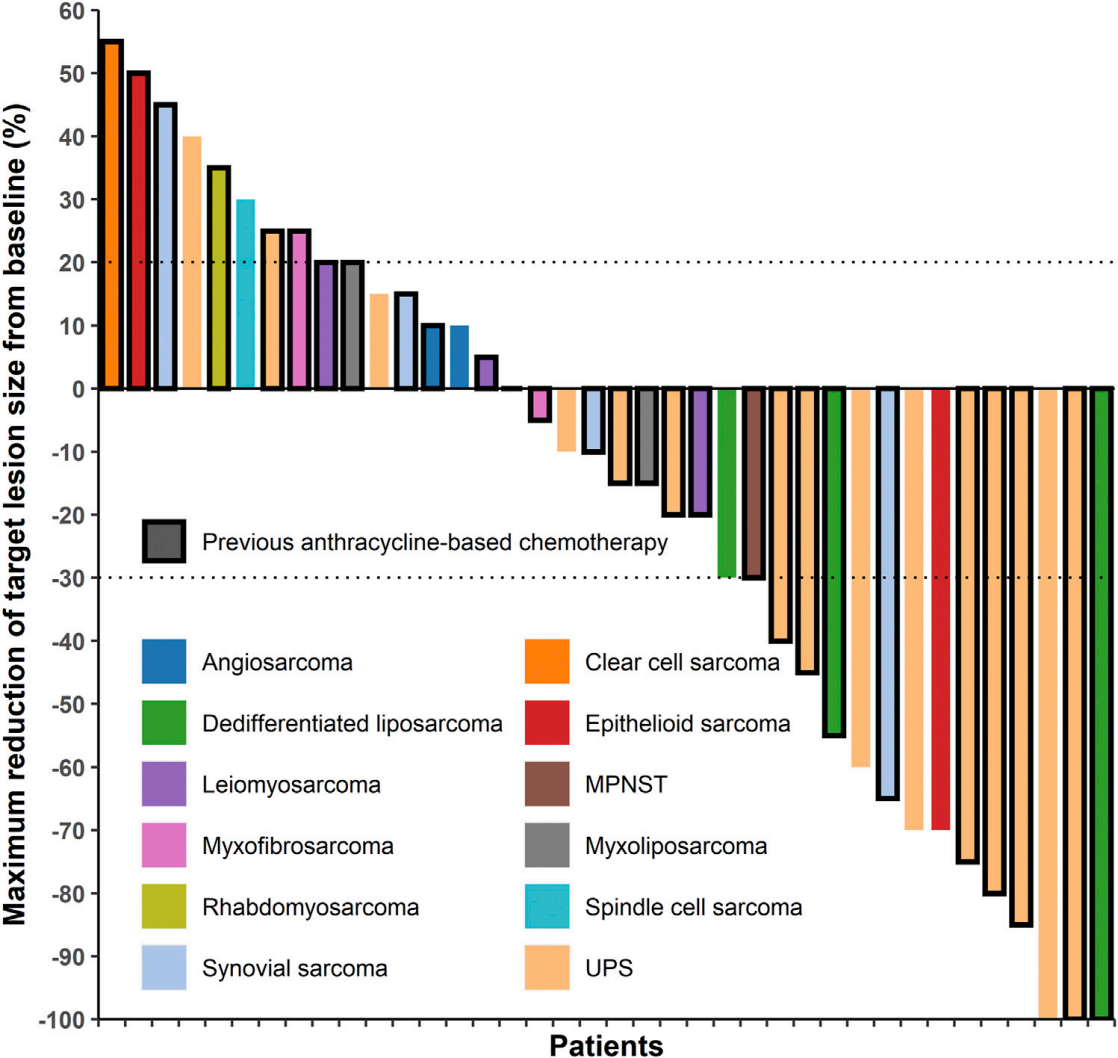
Target lesion changes in patients with soft tissue sarcoma treated with programmed cell death protein one inhibitor plus doxorubicin. The horizontal axis represents different patients and the vertical axis represents the percentage of change in the target lesions. Twenty-eight patients who had received anthracycline-based chemotherapy are marked with a black border. Two patients with undifferentiated pleomorphic sarcoma and one patient with dedifferentiated liposarcoma had a complete response (100% decrease in target lesion size), twelve patients had a partial response (30% and more decrease in target lesion size), twelve patients had stable disease (<20% increase and <30% decrease in target lesion size), and eleven patients had progressive disease (20% and more increase in target lesion size). MPNST, malignant peripheral nerve sheath tumor; UPS, undifferentiated pleomorphic sarcoma.

In the patients with STS included in this study, the response rates of different histological subtypes to sintilimab plus doxorubicin were different (Table 2 and Figure 1). The best response was observed in patients with UPS and dedifferentiated liposarcoma. Among the 16 patients with UPS, two had CR and seven had PR; among the three patients with dedifferentiated liposarcoma, one had CR and two had PR (Table 2 and Figure 1). The overall ORR was 39.5%, DCR was 71.1%, and the median PFS was 4.5 months [95% confidence interval (CI), 3.0–8.5 months] (Table 3).

**TABLE 2:** Best responses of various histological subtypes to treatment.

Histology	Number of patients			
	CR	PR	SD	PD
UPS (n = 16)	2	7	5	2
Synovial sarcoma (n = 4)	0	1	1	2
Dedifferentiated liposarcoma (n = 3)	1	2	0	0
Leiomyosarcoma (n = 3)	0	0	2	1
Myxofibrosarcoma (n = 2)	0	0	1	1
Myxoliposarcoma (n = 2)	0	0	1	1
Angiosarcoma (n = 2)	0	0	2	0
Epithelioid sarcoma (n = 2)	0	1	0	1
Rhabdomyosarcoma (n = 1)	0	0	0	1
MPNST (n = 1)	0	1	0	0
Spindle cell sarcoma (n = 1)	0	0	0	1
Clear cell sarcoma (n = 1)	0	0	0	1
Total	3	12	12	11

Tumor responses were evaluated according to the Response Evaluation Criteria in Solid Tumors (version 1.1), and were categorized as complete response (CR), partial response (PR), stable disease (SD), or progressive disease (PD). Abbreviations: UPS, undifferentiated pleomorphic sarcoma; MPNST, malignant peripheral nerve sheath tumor.

**TABLE 3:** Clinical efficacy in all patients.

Characteristics	Data
ORR (%)	39.5 (95% CI: 24.04–56.61)
DCR (%)	71.1 (95% CI: 54.10–84.58)
M-PFS (months)	4.5 (95% CI: 3.0–8.5)
4 months PFS rate (%)	52.6 (95% CI: 38.9–71.2)
6 months PFS rate (%)	36.8 (95% CI: 24.3–55.9)

Data are presented as percentages or probabilities (95% CI). Abbreviations: ORR, objective response rate; DCR, disease control rate; M-PFS, median progression-free survival; CI, confidence interval.

### Safety

**FIGURE 2:**
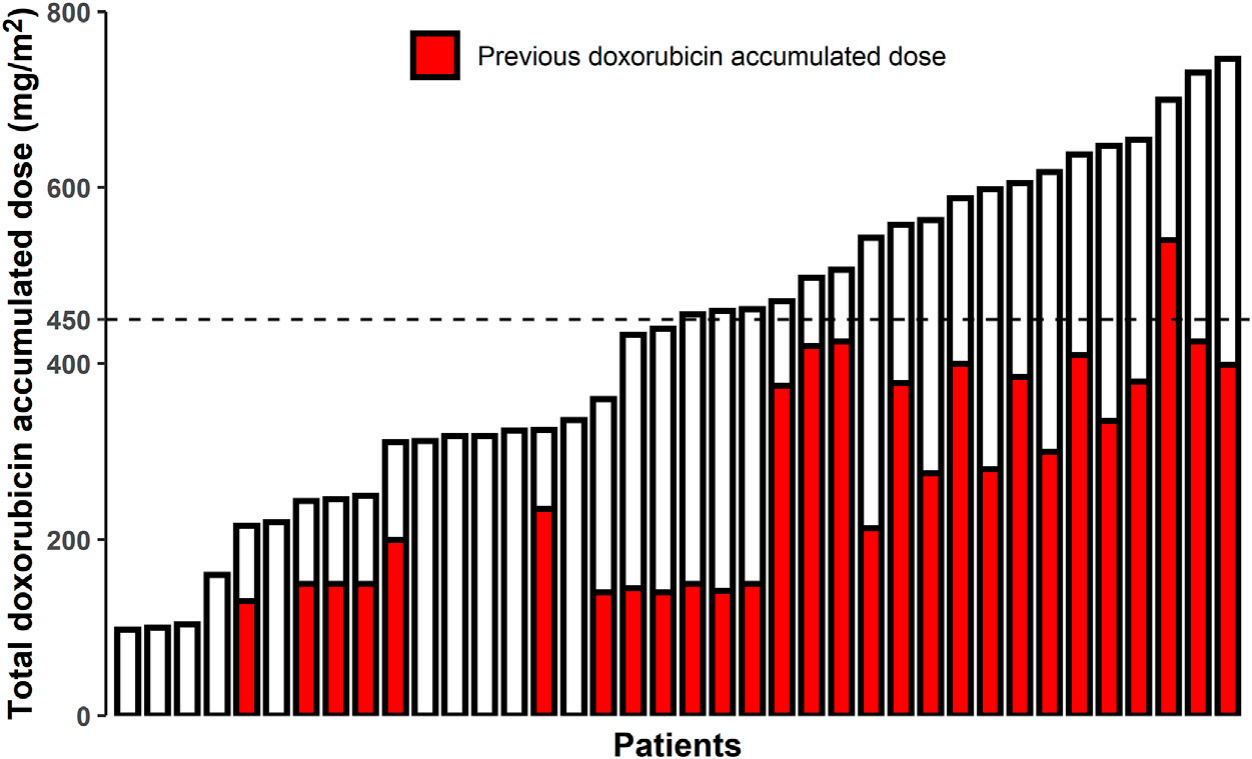
Cumulative doxorubicin dose for patients in this study. Ten patients had not received doxorubicin (or anthracyclines) before the study. The highest total doxorubicin cumulative dose was 747 mg/m^2^. The total doxorubicin cumulative dose exceeded the traditionally recommended upper limit of 450 mg/m^2^ in 19 patients.

As of the data cutoff date, the median number of sintilimab doses delivered was 7 (range 1–24) and that of doxorubicin was 5 (range 2–6). The mean dose of doxorubicin per chemotherapy cycle was 48.5 ± 9.3 mg/m^2^. The total cumulative dose of doxorubicin exceeded 450 mg/m^2^ in 19 patients (Figure 2). Overall, the AEs associated with the combined treatment were mild, manageable, and well-tolerated (Table 4). No symptomatic cardiotoxicity was observed in patients with a total doxorubicin cumulative dose higher than 450 mg/m^2^. However, one patient discontinued treatment because of grade 4 liver injury caused by pembrolizumab and doxorubicin. The most common grade 3 or higher AEs were hematologic and included leukopenia (21.1%), anemia (18.4%), and thrombocytopenia (18.4%) (Table 4). The most common immune-associated AEs associated with sintilimab included immune hepatitis, hypothyroidism, and pneumonitis. As of the data cutoff date, two patients (5.3%) remained on sintilimab therapy, half (19/38) of the patients had died as the primary disease progressed, and the other 17 patients were undergoing other treatments. No treatment-related deaths occurred during this period.

**TABLE 4:** Adverse events.

Adverse Event	All grades	Grade >2
Alopecia	35 (92.1%)	0 (0%)
Leucopenia	34 (89.5%)	8 (21.1%)
Anemia	30 (78.9%)	7 (18.4%)
Fatigue	28 (73.7%)	1 (2.6%)
Thrombopenia	26 (68.4%)	7 (18.4%)
Nausea	20 (52.6%)	0 (0%)
Vomiting	16 (42.1%)	1 (2.6%)
Fever	10 (26.3%)	0 (0%)
Diarrhea	8 (21.1%)	1 (2.6%)
Anorexia	6 (15.8%)	0 (0%)
Transaminase increase	6 (15.8%)	1 (2.6%)
Weight loss	5 (13.2%)	0 (0%)
Hypothyroidism	5 (13.2%)	0 (0%)
Pneumonitis	4 (10.5%)	1 (2.6%)
Cough	3 (7.9%)	1 (2.6%)
Constipation	3 (7.9%)	0 (0%)
Bacteremia	2 (5.3%)	1 (2.6%)
Pain	2 (5.3%)	0 (0%)
Rash	2 (5.3%)	0 (0%)
Pruritus	2 (5.3%)	0 (0%)

Data are presented as numbers (percentages).

### Prognostic analysis

**FIGURE 3:**
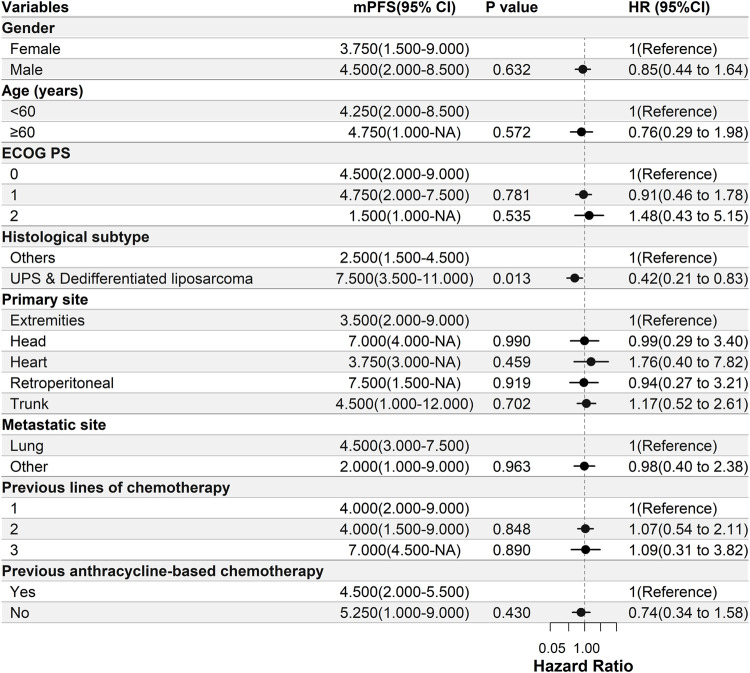
Univariate Cox regression analysis of the relationship between clinicopathological parameters and PFS. Patients with UPS or dedifferentiated liposarcoma have significantly longer PFS than those with other pathological subtypes [hazard ratio (HR) = 0.42, 95% CI 0.21–0.83, *p* = 0.013]. ECOG PS, Eastern Cooperative Oncology Group Performance Status; PFS, progression-free survival; UPS, undifferentiated pleomorphic sarcoma; DOX, doxorubicin; mPFS, median PFS; NA, not available.

**FIGURE 4:**
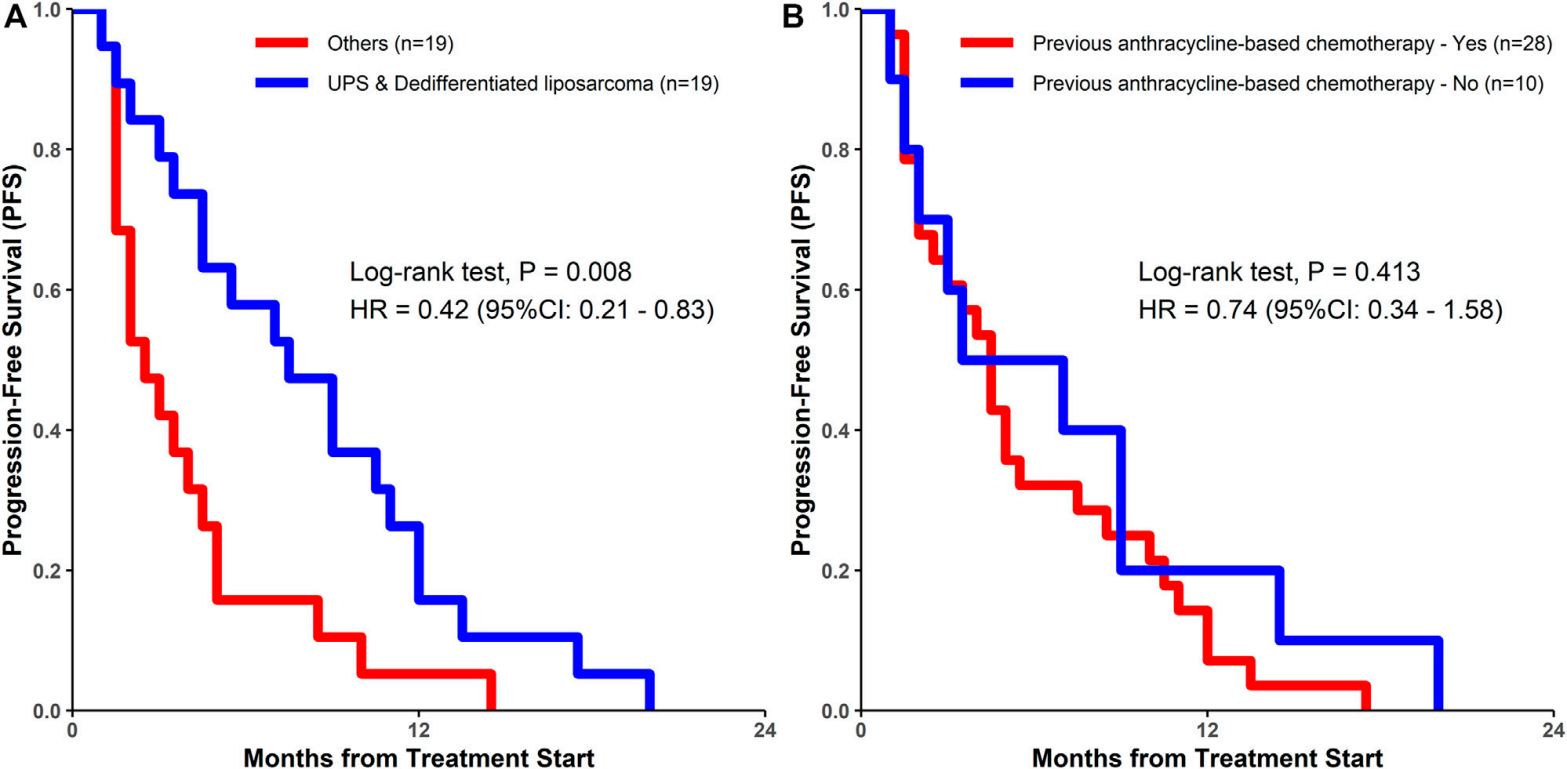
Kaplan-Meier estimates of progression-free survival among **(A)** patients with UPS and dedifferentiated liposarcoma vs. patients with other sarcomas; **(B)** patients who had previously undergone doxorubicin-based chemotherapy vs. those who had not undergone doxorubicin-based chemotherapy. UPS, undifferentiated pleomorphic sarcoma; DOX, doxorubicin; HR, hazard ratio.

Univariate Cox regression analysis was used to test the relationship between the clinical characteristics and prognosis (Figure 3). Only the histological subtypes showed significantly different outcomes among the many clinical features. Patients with UPS or dedifferentiated liposarcoma had a significantly longer PFS than those with other pathological subtypes [hazard ratio (HR) = 0.42, 95% CI 0.21–0.83; *p* = 0.013] (Figures 3, 4). It should be noted that there was no significant difference in the median PFS between patients who had previously received doxorubicin-based chemotherapy and those who had not (HR = 0.74, 95% CI 0.34–1.58, *p* = 0.413) (Table 5; Figures 3, 4).

**TABLE 5:** Clinical efficacy in patients with or without previous anthracycline-based chemotherapy.

Characteristics	Previous anthracycline-based chemotherapy	No	*p*-value
	Yes		
ORR (%)	35.7 (18.6–55.9)	50.0 (18.7–81.3)	0.473
DCR (%)	67.9 (47.6–84.1)	80.0 (44.4–97.5)	0.690
M-PFS (months)	4.50 (2.5–8.5)	5.25 (2.0–NA)	0.413
4 months PFS rate (%)	53.6 (38.0–75.6)	50.0 (26.9–92.9)	0.846
6 months PFS rate (%)	32.1 (18.8–55.1)	50.0 (26.9–92.9)	0.324

Data are presented as percentages or probabilities (95% CI).

## Discussion

Chemotherapy is believed to improve the antitumor effect of PD-1 inhibitors by reducing the number of tumor cells, promoting the death of immunogenic tumor cells, depleting immunosuppressive cells, increasing the number and activity of antitumor immune-effector T cells, and enhancing the secretion of cytokines promoting the proliferation of immune cells (Salas-Benito et al., 2021; Principe et al., 2022). At present, PD-1 inhibitor plus chemotherapy has been approved for the treatment of lung, gastroesophageal, and breast cancers (Wu et al., 2022). This combination therapy has been tested in many other cancers (Principe et al., 2022).

In this open-label, single-center, single-arm phase II trial, the PD-1 inhibitor sintilimab plus doxorubicin was relatively well-tolerated. ORR, DCR, and median PFS were 39.5%, 71.1%, and 4.5 months, respectively. Compared with that noted in previous studies on doxorubicin-based chemotherapy in sarcoma, the efficacy of the combination therapy in this study was significantly improved in UPS and dedifferentiated liposarcoma (McGovern et al., 2017; Seddon et al., 2017). Notably, previous studies have also demonstrated the efficacy of the PD-1 inhibitor plus doxorubicin in UPS and dedifferentiated liposarcoma (Tian et al., 2020a; Pollack et al., 2020; Livingston et al., 2021). This suggests that the PD-1 inhibitor plus doxorubicin may be the most effective treatment for these two sarcoma subtypes. However, we have not been able to determine yet whether the combined regimen is an additive effect or a synergistic effect in these two sarcoma subtypes.

In this study, all the enrolled patients had received other systemic therapy. Some had received anthracycline-based chemotherapy. Our previous study demonstrated that the benefits of using anthracyclines at doses above the upper recommended cumulative dose (450 mg/m^2^) outweighed the risks in some patients (Tian et al., 2020b). Therefore, patients previously administered anthracycline were not excluded in this study. This study is the first to confirm that patients with STS who previously received anthracycline-based chemotherapy can still benefit from a PD-1 inhibitor plus doxorubicin. This is important for many patients with advanced diseases who previously received neoadjuvant chemotherapy. In addition, some patients were previously treated with TKIs. Most of them were administered anlotinib, which is marketed in China. Anlotinib has a median PFS of 5.6 months in STS (Chi et al., 2018; Li, 2021). A future prospective study of this combination regimen and anlotinib is needed to assess its benefits in these patients.

Although in this study, we deliberately chose to administer PD-1 inhibitor 48 h after the completion of chemotherapy, the overall efficacy did not seem to improve compared with that in other studies where PD-1 inhibitor was administered simultaneously with chemotherapy (Tian et al., 2020a; Pollack et al., 2020). This differs from studies that report that the timing of administration can significantly affect the efficacy of PD-1 inhibitors plus chemotherapy (Yao et al., 2021; Zhu et al., 2022). In addition, many factors affect the efficacy of PD-1 inhibitors in combination therapy, such as the timing of administration, use of growth factors, antibiotic use, and *Helicobacter pylori* infections (Cortellini et al., 2020; Salas-Benito et al., 2021; Oster et al., 2022). More in-depth and extensive studies are needed to improve the efficacy of PD-1 inhibitors plus doxorubicin in STSs.

Sintilimab plus doxorubicin has a better safety profile than doxorubicin plus ifosfamide (Judson et al., 2014). The high safety profile allows for further testing of combination therapy in patients with poor physical fitness or in those who are older. The reason for the high safety in this study may be that the doxorubicin dose was lower than the conventional dose. An overdose of chemotherapeutic drugs inevitably leads to severe immunosuppression, weakening the efficacy of PD-1 inhibitors. Therefore, the dose of chemotherapy used in chemoimmunotherapy should be minimized. However, low doses of chemotherapeutic agents might not necessarily produce synergistic effects with PD-1 inhibitors (Lin et al., 2022). Hence, the optimal dose estimation of doxorubicin in combination with PD1 inhibitors requires further studies. It should be noted that some patients in this study used doxorubicin at a super-cumulative dose. No symptomatic cardiotoxicity was observed in these patients. This is similar to the results of some other studies on the use of doxorubicin at super-cumulative doses (Tian et al., 2020b). Therefore, the combined regimen has acceptable safety in patients who previously received anthracycline-based therapy. However, the cumulative dose of doxorubicin exceeded the upper limit of the traditional recommended dose, and it is important to monitor cardiac adverse reactions in these patients in future studies.

The results of this study provide important reference values. First, this study confirmed that patients with advanced STS who had previously received anthracycline-based chemotherapy could still benefit from PD-1 inhibitor plus doxorubicin treatment. Second, the efficacy of this combination therapy was significantly higher in patients with UPS and dedifferentiated liposarcoma than in those with other sarcomas. The main shortcomings of this study were the lack of a control group, limited number of cases, and that it was done in a single center. The lack of routine records of cardiac adverse reactions such as left ventricular ejection fraction after the ultra-cumulative doxorubicin use was also a major shortcoming of this study. In future research, a study with a larger number of patients with UPS and dedifferentiated liposarcoma should be performed, and the specific protocol of the combination therapy in patients with UPS and dedifferentiated liposarcoma should be further studied.

In conclusion, sintilimab plus doxorubicin is a safe and promising treatment for patients with advanced STS after the failure of previous systemic therapy. The efficacy of this combination therapy is significantly higher in patients with UPS and dedifferentiated liposarcoma than in those with other sarcomas and deserves further study in an extended clinical trial.

## Data Availability

The raw data supporting the conclusion of this article will be made available by the authors, without undue reservation.

## Abbreviations

ORR, The objective response rate; DCR, The disease control rate; M-PFS, median progression-free survival;CI, confidence interval; NA, not available.
